# Characterization of myocardium and myocardial motion in patients considered for transaortic valve implantation (TAVI)

**DOI:** 10.1186/1532-429X-15-S1-P113

**Published:** 2013-01-30

**Authors:** Christopher Schneeweis, Bernhard Schnackenburg, Christian T Stoeck, Alexander Berger, Thomas Hucko, Sebastian Kelle, Daniel Messroghli, Eckart Fleck, Rolf Gebker

**Affiliations:** 1 Cardiology, German Heart Institute, Berlin, Germany; 2 Clinical Science, Philips Health Care, Hamburg, Germany; 3 Institute for Biomedical Engineering, University and ETH Zürich, Zürich, Switzerland; 4 Congenitial Heart Disease, German Heart Defects, Berlin, Germany

## Background

Aortic valve stenosis (AS) is a common disease. In older patients open chest valve replacement is limited due a high periprocedural risk. Therefore transaortic valve implantation (TAVI) has become a potential alternative treatment. AS causes alterations of cardiac rotation and torsion and triggers diastolic dysfunction. A poorer prognosis was observed in the presence of myocardial fibrosis (MF). Cardiac magnetic resonance imaging (CMR) can depict myocardial fibrosis by using the late gadolinium enhancement (LGE) technique. Recently T1-mapping showed promising results for the characterization of myocardial tissue. Aim of this study was to characterize the myocardium functionally and morphologically.

## Methods

A total amount of 30 patients (16 females, age 80.6 ± 5.8 years) with severe AS were considered for TAVI. CMR was performed using a clinical 1.5T MR system (Philips Achieva). Cine imaging was performed using short and long axis orientation. Tagging of three short axis planes (apical, medial, basal) was acquired using the CSPAMM. T1 mapping of the medial short axis slice was performed prior and 10 minutes after contrast application. Quantification of global MF was assessed by using the short-axis LGE images with a threshold of ± 2 SD above a normal region defining MF. LGE images were acquired 10-15 minutes after bolus injection of 0.2 mmol/kg gadoterate meglumine.

## Results

We observed an averaged normal LVEF with increased myocardial mass (125 ± 41 g). Fibrotic tissue was evaluated in 20 patients with a total amount of 3.6 ± 4.6%. The extracellular volume fraction (ECV) as assessed from T1 pre- and post T1 maps and hematocrit was higher (range: 23.8 - 57.9% with mean 31.6 ± 8.2%) than previously published in healthy tissue and in agreement with data in AS. Significant differences of tagging parameters were observed between the basal and apical region (circumferential strain basal: -15.2 ± 3.6% vs. apical: -18 ± 3.3 % with p <0.001; rotation basal: 5.3 ± 3.3° vs. apical: 18 ± 4.9° with p <0.001; systolic peak rotation velocity basal: 38.7 ± 14.8 °/s vs. apical: 84.8 ± 21.6°/s with p<0.001; diastolic peak rotation velocity basal: -52.1 ± 21.6 vs. apical: 127.7 ± 27.4 °/s). Furthermore a reduced diastolic strain rate was evaluated (0.42 ± 0.25s^-1^).

## Conclusions

Our findings of significant differences between apical and basal rotation are in agreement with prior studies. The systolic and diastolic rotation velocity was significantly higher in the apical region and may be caused by severe pressure overload. In relation to published data, our patients showed a reduced diastolic strain rate, which serves as advise for diastolic dysfunction.

## Funding

no funding

